# Patient-reported outcome assessment of adults and adolescents with atopic dermatitis: a cross-sectional qualitative interview study

**DOI:** 10.1186/s41687-025-00871-8

**Published:** 2025-04-10

**Authors:** Parima Ghafoori, Dharm S. Patel, Kimberly Raymond, Elizabeth Brennan, April Mitchell Foster, Kristi Jackson, Helen J. Birch, Wen-Hung Chen

**Affiliations:** 1 GSK, 1250 South Collegeville Rd, Collegeville, PA 19426 USA; 2 QualityMetric Incorporated, Johnston, RI USA; 3 GSK, London, UK

**Keywords:** Atopic dermatitis, Patient-reported outcome measures, Fatigue, Quality of life, Sleep disturbance, Skin pain, Qualitative study

## Abstract

**Background:**

Atopic dermatitis (AD) is a chronic inflammatory skin disease that impacts patient health and quality of life. Understanding patient experience of relevant symptoms and impacts of AD is crucial for improving outcomes. This study aimed to characterise adult (≥ 18 years) and adolescent (12–17 years) patients’ experiences of AD and assess the content validity of selected patient-reported outcomes (PROs).

**Methodology:**

This non-interventional, cross-sectional, qualitative study recruited US-based, English-speaking adults and adolescents with moderate-to-severe AD, either naïve or experienced with biologics. A 90-minute interview was conducted via teleconferencing software, consisting of concept elicitation (CE) of AD experiences and cognitive debriefing (CD), where participants provided feedback on PROs assessing skin pain, sleep disturbance, and fatigue. Interview data were coded and analysed using qualitative data software to determine the AD experience and content validity of selected PROs. A conceptual disease model was developed from the CE portion of the interview. Results from the CD portion were mapped to this model to evaluate the conceptual coverage of the PROs.

**Results:**

In total, 16 adults (mean age 48 years, 56% White, 63% female, 50% biologic naïve) and 20 adolescents (mean age 16 years, 60% White, 75% female, 50% biologic naïve) were included in the analysis. During CE, 13 symptoms and impacts in 7 domains were identified. The most reported symptom was itchiness (adults, 100%; adolescents, 100%) and the most reported impact was emotional functioning (adults, 94%; adolescents 95%). Participants also commonly reported experiencing pain/discomfort (adults, 69%; adolescents, 80%) and sleep disturbance (adults, 88%; adolescents, 75%). Fatigue was reported by 94% of adults across CE and CD segments. When probed during CE, 65% of adolescents identified fatigue as an impact of AD. During CD, 70–100% of participants confirmed the selected PROs were comprehensible and relevant.

**Conclusions:**

This study provides evidence that the experience of AD is similar between adults and adolescents as well as biologic-naïve and biologic-experienced participants. Relevant disease concepts in patients with AD were identified, and content validity was established for the selected PROs, supporting their use in future clinical studies.

**Supplementary Information:**

The online version contains supplementary material available at 10.1186/s41687-025-00871-8.

## Background

Atopic dermatitis (AD) is a chronic inflammatory skin condition, characterised by pruritus, dry skin, and eczematous lesions, affecting approximately 5–20% of children and 18–20% of adults worldwide [1, 2]. AD symptoms can manifest anywhere on the body, leading to a substantial impact on patients’ health-related quality of life (HRQoL), with disease severity correlating with HRQoL impairment [2–4]. Itch, redness, dry skin, and pain are common AD symptoms, often leading to impacts such as sleep disturbance, fatigue, and emotional distress (including depression and anxiety) [3, 5, 6].

Although topical moisturisers and corticosteroids have been used to manage moderate-to-severe AD symptoms [7], advanced therapy options, such as biologics and small molecule inhibitors, including Janus kinase inhibitors, have enhanced the treatment landscape for patients with moderate-to-severe AD in the United States (US) [7, 8]. As the treatment landscape evolves, it is important to assess ongoing symptoms and impacts of AD, particularly for patients treated with recently available advanced therapies.

Understanding the disease burden from the patients’ perspectives via patient-reported outcomes (PROs) is crucial for improving clinical outcomes and HRQoL [3]. Recent clinical and Food and Drug Administration (FDA) guidelines emphasise the importance of including PROs in clinical studies, focusing on concepts important to patients with AD, such as itch, sleep disturbance, and HRQoL [8–10]. Itch and skin pain have been shown to cause sleep disturbance in patients with AD, which can lead to fatigue and reduced HRQoL [11, 12]. Clinical practice guidelines emphasise that sleep disturbance and its associated consequences are key measures of disease impact and highlight gaps in validation and uniformity of HRQoL measurements [13].

Fatigue, a common result of sleep disturbance, has been reported 2–3 times more frequently in patients with AD compared with healthy controls [14], yet few studies have assessed patients’ experience of fatigue, necessitating further exploration in the context of AD [6, 12, 14]. Functional Assessment of Chronic Illness Therapy-Fatigue (FACIT-Fatigue) Scale [15] and Brief Fatigue Inventory-item 3 (BFI-item 3) [16] are examples of measures that assess fatigue and have been used in other medical conditions [16, 17]. However, content validity of each PRO needs to be evaluated specifically for patients with AD before their application in clinical studies [9, 18].

Given the evolving treatment landscape and the significant role of symptoms, like skin pain, and impacts, like sleep disturbance and fatigue, on HRQoL, it is essential to include appropriate PROs in clinical trials to assess these aspects in the context of AD. Although AD is a well-studied condition, concept elicitation (CE) remains critical to evaluate the content validity of PROs specific to patients with moderate-to-severe AD and in a biologic-experienced population. The current study aimed to explore the overall experience of AD via CE of adults and adolescents with moderate-to-severe AD, with participants stratified based on previous biologic experience. Furthermore, this study aimed to evaluate the content validity of selected PRO measures for skin pain, sleep disturbance, and fatigue via cognitive debriefing (CD).

## Methods

### Study design

This non-interventional, cross-sectional, qualitative study assessed adult (≥ 18 years of age) and adolescent participants (12–17 years of age) with moderate-to-severe AD from the US. An overview of the study design is presented in Supplementary Figure S1. Ethics approval for this qualitative study was granted by WIRB-Copernicus Group (WCG) − 36561371.0. WCG reviewed and approved all qualitative study materials prior to the start of recruitment.

All adults completed the study before adolescent participant enrolment with some study design amendments being implemented based on the findings for the adult cohort (detailed where relevant below). The 90-minute interview, conducted via teleconferencing software (with caregivers present if desired for adolescent participants), followed a semi-structured interview guide and consisted of CE and CD segments. The study-specific interview guide was developed in line with FDA guidelines [9] (Supplementary Table S1).

### Recruitment and eligibility

Potential participants were screened by telephone via two third-party recruitment vendors. Eligibility criteria for adult and adolescent participants included: US-based, able to speak and read English, able to provide confirmation of physician-diagnosed AD, moderate-to-severe AD within the past 2 years (self-reported on the day of screening), and able to participate in a 90-minute interview. Participants were asked to confirm prescription for either a biologic or an oral corticosteroid (within the past 2 years), which was used as a proxy for the participant having experienced moderate-to-severe symptoms. The study aimed to recruit equal numbers of biologic-naïve and biologic-experienced participants in both adult and adolescent cohorts.

### CE and conceptual model development

During CE, a combination of pre-set open-ended questions and ad hoc probing was used by researchers to elicit descriptions of the participant’s symptoms, impacts, and overall experience with AD. Based on interview findings, a conceptual disease model was developed to visually represent the participant’s experience of AD, including symptoms and impacts.

### CD and conceptual mapping

For the CD aspect of the interview, researchers used a combination of a think-aloud method and pre-set open-ended questions to evaluate PRO measures in terms of relevance, comprehensibility, and comprehensiveness.

The selected concepts and PROs were: sleep disturbance, measured by Patient-Reported Outcomes Measurement Information System—Sleep Disturbance (PROMIS-SD) Short Forms 8a/8b [19] and PROMIS Pediatric-SD 8a (peds PROMIS-SD 8a) [20]; fatigue, measured by BFI-item [3, 16], FACIT-Fatigue [15], and Pediatric FACIT-Fatigue (peds FACIT-Fatigue) [21]; and skin pain, measured by Skin Pain—Numeric Rating Scale (SP-NRS) [22]. Skin pain was also assessed using pain measures, Patient Global Impression of Severity (PGIS) [23], and Patient Global Impression of Change (PGIC) [23]. Further details of instrument assessment are provided in Supplementary Figure S1. Half of the adolescent participants debriefed the PROMIS-SD Short Forms 8a/8b and FACIT-Fatigue, and half debriefed the peds PROMIS-SD 8a and peds FACIT-Fatigue. All adolescent participants were debriefed with adult versions of BFI-item 3, SP-NRS, PGIS, and PGIC.

Conceptual mapping was used to demonstrate how well each PRO measure addressed targeted concepts related to experience of AD. Two researchers independently mapped the concepts included in the model to the measures. Results were compared and a consensus was reached via discussion. Exemplary participants’ quotes were then mapped to each item in the PRO measures to confirm relatedness.

### Analysis

After each interview, interviewers recorded initial observations of emerging themes using field notes, which were used to develop a coding structure.

The CE interview segment of the transcript was coded and analysed by thematic and content analysis, using NVivo qualitative software, and concepts were assigned to an existing coding framework (developed in advance from questions in the interview guide). In addition to coding to this existing framework, concepts elicited from participants during the interview that were not included in the existing framework were also coded. These codes were developed and refined in an on-going manner as the transcripts were reviewed and analysed [24]. For CD data, Microsoft Excel was used to record any notable issues that arose during the interview (e.g., confusing or unclear items) or suggested changes to the PROs (e.g., recommendations for improved wording), which allowed for the identification of issues or suggestions endorsed by multiple participants. Relevant data from the CD segment was then coded using NVivo qualitative software and analysed along with the conceptual coding of the CE data.

Categorical variables were summarised as numbers and percentages, and continuous variables were summarised as means and ranges.

Sample saturation was conducted to ensure sufficient sample size; concepts/themes identified were tabulated, transcripts were organised into groups of 4 chronologically, and saturation was achieved when no new themes of interest emerged [25–27].

## Results

### Participant demographics and characteristics

A total of 16 adults and 20 adolescents completed the study, which consisted of equal numbers of the two prespecified treatment groups: biologic-naïve (*N* = 8, adults; *N* = 10, adolescents) and dupilumab-treated (*N* = 8, adults; *N* = 10, adolescents).

Mean participant age was 48 years (range 23–61) for adults and 16 years (range 13–17) for adolescents, and over half of participants identified as Caucasian or White (adults, 56% [*n* = 9]; adolescents, 60% [*n* = 12]) and female (adults, 63% [*n* = 9]; adolescents, 75% [*n* = 15]). Baseline characteristics and self-reported AD severity are reported in Table 1 and Supplementary Table S2, respectively.

**Table 1:** Baseline characteristics

	Adults			Adolescents		
	Biologic-naïve (*N* = 8)	Dupilumab- experienced (*N* = 8)	Total (*N* = 16)	Biologic-naïve (*N* = 10)	Dupilumab- experienced (*N* = 10)	Total (*N* = 20)
**Age (years), mean (range)**	45 (23–61)	50 (44–57)	48 (23–61)	16.0 (13–17)	15.6 (13–17)	15.8 (13–17)
**Gender**,^**a**^** n (%)**						
Male	2 (25)	3 (38)	5 (31)	3 (30)	2 (20)	5 (25)
Female	5 (63)	5 (63)	10 (63)	7 (70)	8 (80)	15 (75)
I do not wish to answer	1 (13)	n/a	1 (6)	n/a	n/a	n/a
**Education**,** n (%)**						
Some high school or less	n/a	n/a	n/a	10 (100)	8 (80)	18 (90)
High school graduate	n/a	n/a	n/a	n/a	1 (10)	1 (5)
Trade or vocational school graduate	1 (13)	1 (13)	2 (13)	n/a	1 (10)	1 (5)
Some college	3 (38)	n/a	3 (19)	n/a	n/a	n/a
Associate’s degree	2 (25)	1 (13)	3 (19)	n/a	n/a	n/a
Bachelor’s degree	1 (13)	6 (75)	7 (44	n/a	n/a	n/a
Graduate degree or higher	1 (13)	n/a	1 (6)	n/a	n/a	n/a
**Race and ethnicity**,** n (%)**						
Black/African American	1 (13)	1 (13)	2 (13)	2 (20)	2 (20)	4 (20)
Asian	n/a	n/a	n/a	n/a	1 (10)	1 (5)
Caucasian or White	4 (50)	5 (63)	9 (56)	7 (70)	5 (50)	12 (60)
Mixed race	1 (13)	1 (13)	2 (13)	1 (10)	1 (10)	2 (10)
Hispanic or Latino	1 (13)	n/a	1 (6)	1 (10)	n/a	1 (5)
I do not wish to answer	1 (13)	1 (13)	2 (13)	n/a	n/a	n/a

^a^This study included two separate screening questions for reporting gender and sex; all adults and adolescents reported their gender to be the same as their sex

N/a, Non-applicable

### Concept elicitation and conceptual disease model

A total of 42 and 44 concepts were included in the saturation analysis for adults and adolescents, respectively (Supplementary Table S3). After 8 adult and 16 adolescent interviews, no new concepts emerged, indicating sample saturation was reached.

Triggers of AD were similar between adults and adolescents (Supplementary Table S4) and, for both adults and adolescents, symptoms were experienced in multiple body locations, with the upper (adults, 88% [*n* = 14]; adolescents, 95% [*n* = 19]) and lower (adults: 81% [*n* = 13]; adolescents: 80% [*n* = 16]) extremities being the most reported site of AD (Supplementary Figure S2).

**Fig. 1:**
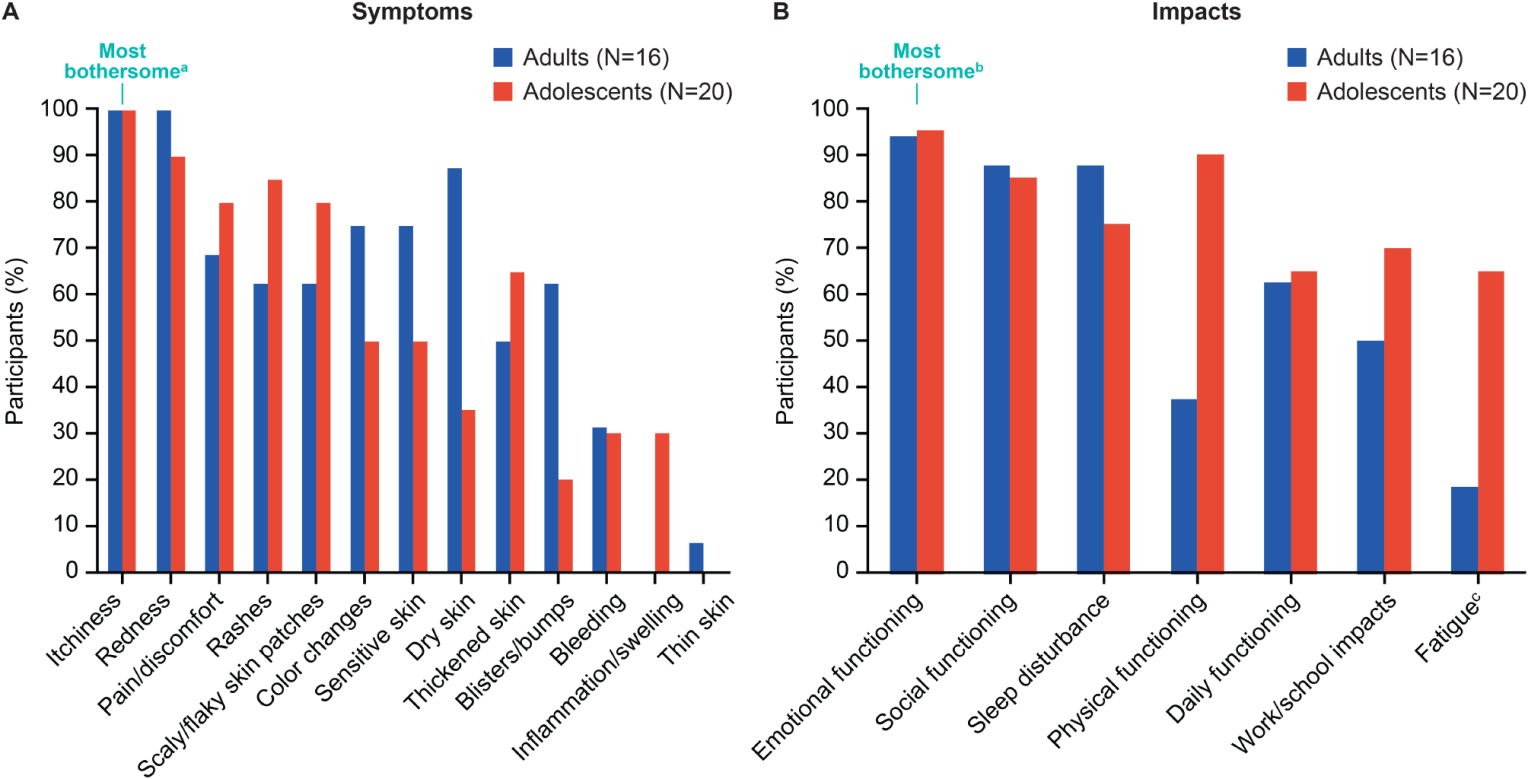
Reported AD (**A**) symptoms and (**B**) impacts. ^a^A total of 14 adults and 19 adolescents reported a most bothersome symptom, and itchiness was most frequently identified by both adults (100% [n=14]) and adolescents (63% [n=12]). ^b^A total of 13 adults and 18 adolescents reported a most bothersome impact domain, and emotional functioning was most frequently identified by both adults (54% [n=7]) and adolescents (56% [n=10]). ^c^For fatigue, data were taken from both CE and CD segments of the interview. When data from both CE and CD segments were considered, 94% of adults (n=15) considered fatigue to be an impact. Based on findings from the adult interviews, adolescent participants were probed on fatigue during CE. AD, atopic dermatitis; CD, cognitive debriefing; CE, concept elicitation.

A total of 13 AD symptoms were identified, with itchiness reported most frequently by both adults (100%, [*n* = 16]) and adolescents (100%, [*n* = 20]) (Fig. 1A). Pain/discomfort was also commonly reported by adults (69% [*n* = 11]) and adolescents (80% [*n* = 16]). Of participants who specified a most bothersome symptom (adults, *N* = 14; adolescents, *N* = 19), itchiness was most frequently identified by both adults (100% [*n* = 14]) and adolescents (63% [*n* = 12]). A total of 4 adults and 4 adolescents reported more than one symptom as most bothersome.

Participants reported impacts in 7 domains: emotional functioning, sleep disturbance, fatigue, social functioning, daily functioning, work/school impacts, and physical functioning (Fig. 1B and Supplementary Table S5). Emotional functioning was the most frequently reported impact domain by both adults (94%, [*n* = 15]) and adolescents (95%, [*n* = 19]). In addition, most participants reported sleep disturbance (adults, 88% [*n* = 14]; adolescents, 75% [*n* = 15]), including difficulty staying asleep (adults, 69% [*n* = 11]; adolescents, 50% [*n* = 10]) and falling asleep (adults, 44% [*n* = 7]; adolescents, 75% [*n* = 15]) (Supplementary Table S5). Of participants who specified a most bothersome impact domain (adults, *N* = 13; adolescents *N* = 18), emotional functioning was most frequently identified by both adults (54% [*n* = 7]) and adolescents (56% [*n* = 10]). One adult and one adolescent reported two most bothersome impacts.

Adult participants were not explicitly probed on fatigue during CE and only a single participant mentioned fatigue spontaneously; however, most participants described experiencing fatigue during CD. When data from both CE and CD segments were considered, 94% of adults (*n* = 15) considered fatigue to be an impact. Data were, therefore, taken from both the CE and CD segments of the interview for the adult cohort to provide a more comprehensive picture of fatigue. Based on this finding, the study design was amended for adolescents to allow researchers to probe participants about fatigue during CE. Approximately two-thirds of adolescents (65% [*n* = 13]) reported fatigue as an impact of AD when probed (Fig. 1B and Supplementary Table S5). Most participants noted they had not considered fatigue a clinical aspect or symptom of their AD. Sleep disturbances, driven by nocturnal scratching and skin picking, had been perceived as the primary cause of fatigue. Adults and adolescents, however, attributed their fatigue to different sources. The same proportion of adult participants attributed fatigue to sleep disruption (44% [*n* = 7]) or stress, anxiety, and embarrassment (44% [*n* = 7]), whereas one participant (6%) attributed fatigue to AD flares. By contrast, 50% (*n* = 10) of adolescents attributed fatigue to sleep disruption, whereas 20% (*n* = 4) attributed fatigue to stress, anxiety, and embarrassment and 15% (*n* = 3) attributed fatigue to AD flares.

Despite general similarities in the reported AD impacts between adults and adolescents, a notable difference was observed between the proportion of adults and adolescents reporting an impact on physical functioning (adults, 37% [*n* = 6]; adolescents, 90% [*n* = 18]) (Supplementary Table S5). Most adolescents changed their hygiene routine (80% [*n* = 16]) and half avoided or limited exercise (50% [*n* = 10]), compared with 19% (*n* = 3) and 25% (*n* = 4) of adults, respectively.

For both adults and adolescents, the experience of AD did not differ by treatment history, with both biologic-naïve and -experienced participants reporting similar AD symptoms and impacts (including itch, sleep disturbance, and fatigue) (Supplementary Table S5).

**Fig. 2:**
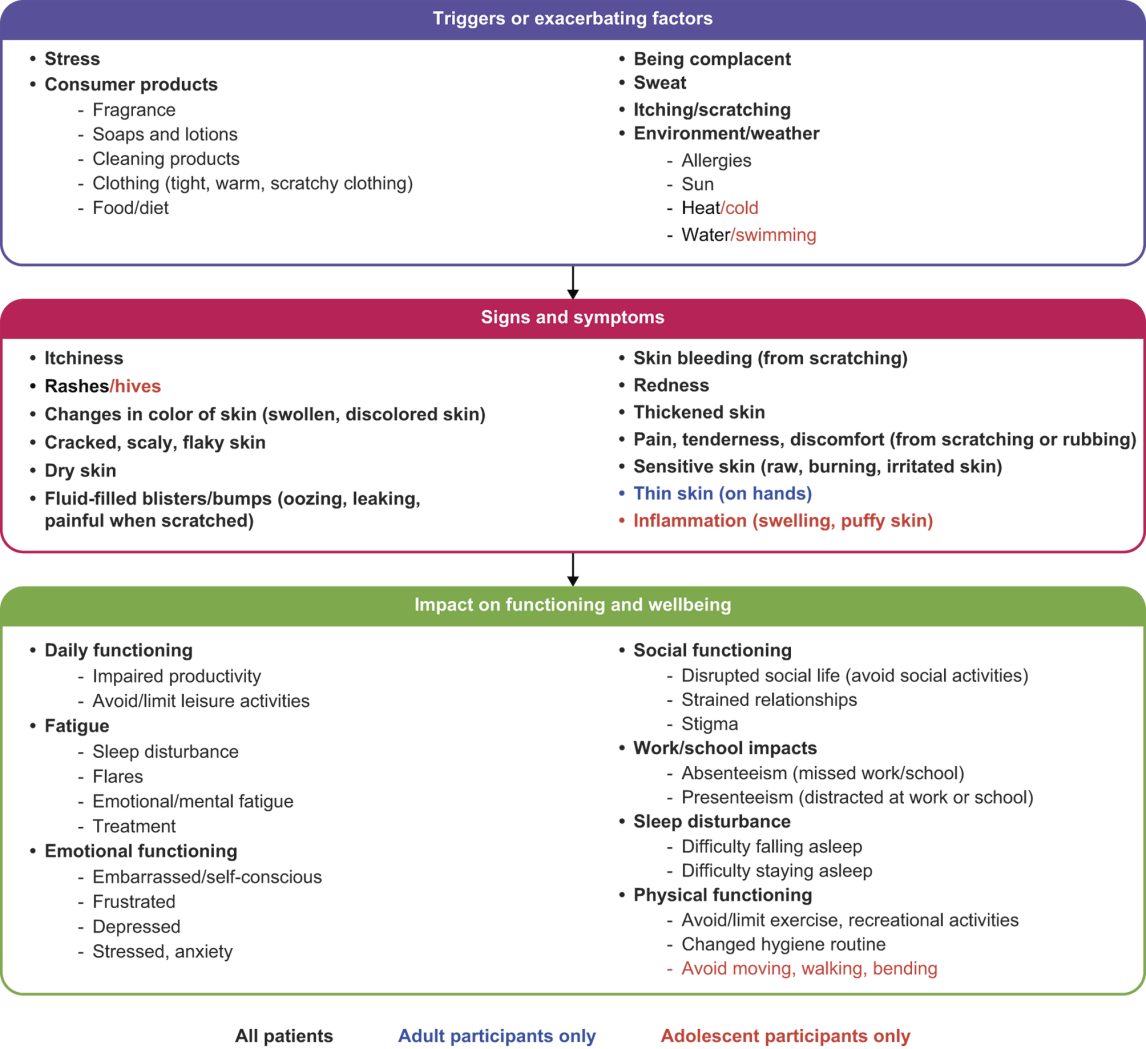
Conceptual disease model of AD. AD, atopic dermatitis

Key concepts identified during CE were used to develop a conceptual disease model (Fig. 2).

### Cognitive debriefing

**Fig. 3:**
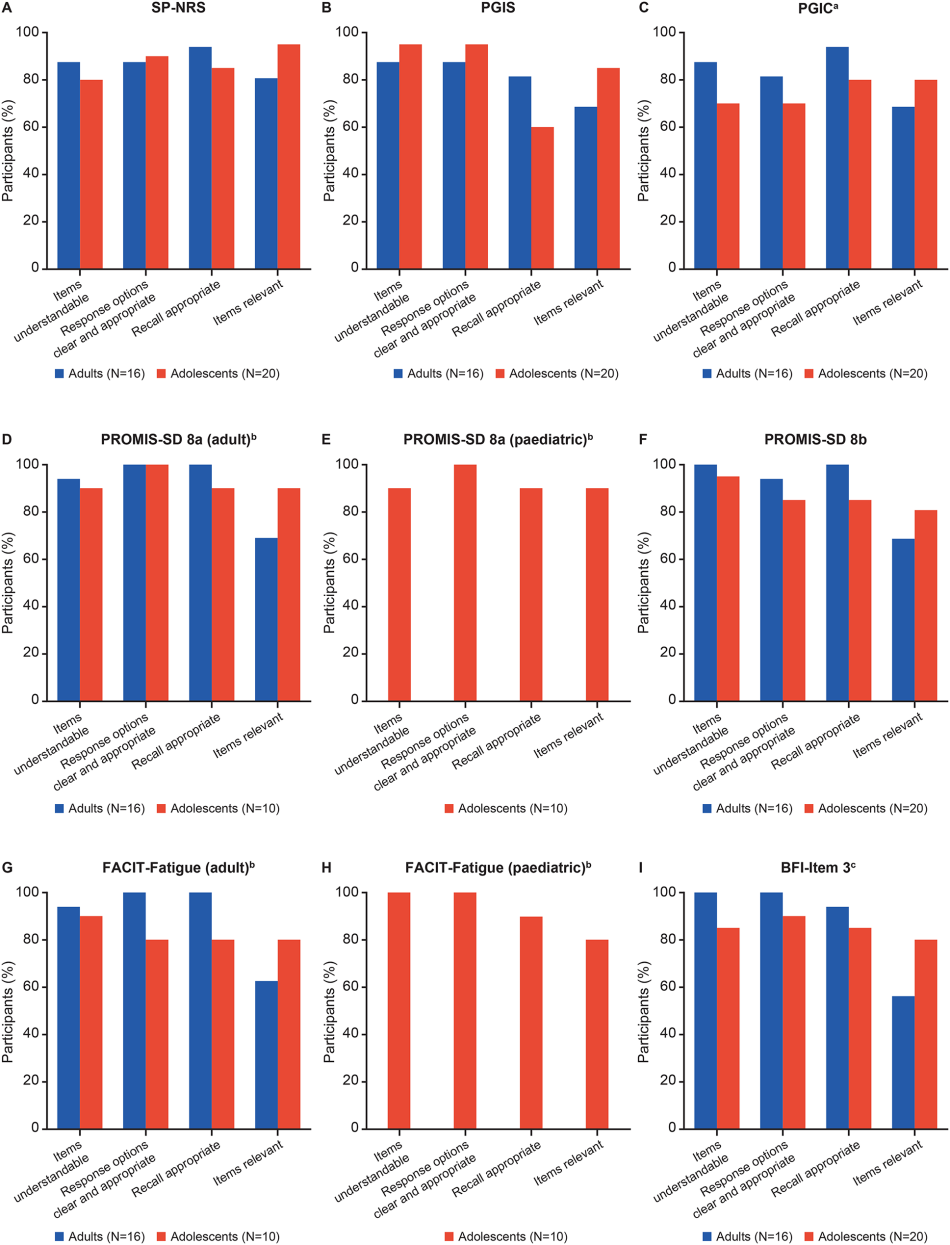
CD of (**A**) SP-NRS, (**B**) PGIS, (**C**) PGIC, (**D**) PROMIS-SD 8a, (**E**) Peds PROMIS-SD 8a, (**F**) PROMIS-SD 8b, (**G**) FACIT-Fatigue, (**H**) Peds FACIT-Fatigue, and (**I**) BFI-Item 3.^a^For PGIC, adolescents reported challenges with the response options, with 4 adolescents feeling there were too many choices, and 3 adolescents preferring a shorter recall period. ^b^Results based on smaller sample size of n=10 for the adolescent group, as the group was divided to complete the adult and paediatric forms of the questionnaire. ^c^For BFI-Item 3, 7 adults reported that BFI-item 3 was not relevant to AD, with 4 adults finding it difficult to see a connection between a skin condition and fatigue. AD, atopic dermatitis; BFI-item 3, Brief Fatigue Inventory-item 3; CD, cognitive debriefing; FACIT-Fatigue, Functional Assessment of Chronic Illness Therapy-Fatigue; peds FACIT-Fatigue, Pediatric Functional Assessment of Chronic Illness Therapy-Fatigue; peds PROMIS-SD 8a, Patient-Reported OutcomesMeasurement Information System—Pediatric Sleep Disturbance; PGIC, Patient Global Impression of Change; PGIS, Patient Global Impression of Severity; PRO, patient-reported outcome; PROMIS-SD, Patient-Reported Outcomes Measurement Information System—Sleep Disturbance; SP-NRS, Skin Pain Numerical Rating Scale

Concepts measured by the selected PROs were deemed important to patients’ experience with AD. Both adult and adolescent participants reported mostly positive feedback, with 81–100% of adults and 70–100% of adolescents stating that PRO measures were comprehensible (Fig. 3).

Most participants reported skin pain measures as relevant to their experience of AD: SP-NRS (adults, 81% [*n* = 13]; adolescents, 95% [*n* = 19]), PGIS for skin pain (adults, 69% [*n* = 11]; adolescents, 85% [*n* = 17]), and PGIC for skin pain (adults, 69% [*n* = 11]; adolescents, 80% [*n* = 16]) (Fig. 3). Additionally, 69% of adults (*n* = 11) confirmed the content validity of sleep disturbance measure PROMIS-SD 8a/b and 90% (*n* = 18) and 80% (*n* = 16) of adolescents confirmed the content validity of the PROMIS-SD 8a and 8b, respectively (Fig. 3d & e). Adolescents stated that the PROMIS-SD 8a/b was especially relevant for capturing the increased levels of sleep disturbance experienced with more severe AD symptoms, as disruption to sleep was mostly attributed to itchiness.

When considering fatigue PRO measures, 63% of adults (*n* = 10) found FACIT-Fatigue to be relevant to their experience of AD and, although 38% of adults (*n* = 6) were uncertain, upon further probing, they noted experiences of fatigue from stress or sleep disturbance related to AD. Additionally, most adolescents (80% [*n* = 8] for FACIT-Fatigue and 80% [*n* = 8] for ped FACIT-Fatigue) reported that fatigue items were relevant to their AD experience (Fig. 3g & h). Over half of adults (56% [*n* = 9]) and most adolescents (80% [*n* = 16]) confirmed the content validity of BFI-item 3, especially when their AD was more severe (Fig. 3i and Supplementary Table S6).

No difference was reported by adolescents in the relevance, appropriateness, and understandability of the PROMIS-SD 8a (*N* = 10) versus the peds PROMIS-SD 8a (*N* = 10); a slightly higher proportion of adolescent participants who debriefed the ped FACIT-Fatigue (*N* = 10) reported that instructions of the paediatric version were clear and easy to understand (100% [*n* = 10]) compared with those who debriefed the FACIT-Fatigue (90%, [*n* = 9]).

### Conceptual mapping

Conceptual mapping demonstrated that the seven selected PRO measures provided coverage of the specific concepts of interest, mainly sleep disturbance, fatigue, and skin pain (Table 2). The SP-NRS, PGIS for skin pain, and PGIC for skin pain measures covered severity and change associated with skin pain, tenderness, and discomfort resulting from symptoms of AD (Table 2).

**Table 2:** Breakdown of AD conceptual coverage of pros for adults and adolescents

Concept	PROMIS-SD 8a	PROMIS-SD 8b	BFI-item 3	FACIT-Fatigue	SP-NRS	PGIS	PGIC
**S** **ymptoms**							
**Itchiness**							
**Rashes** ***/*** **hives** ^**a**^							
**Changes in color of skin**							
**Dry skin**							
**Fluid-filled blisters/bumps**							
**Skin bleeding**							
**Redness**							
**Thickened skin**							
**Pain**,** tenderness**,** discomfort**					**x**	**x**	**x**
**Sensitive skin**							
**Inflammation (swelling**,** puffy skin)**^**a**^							
**Thin skin**							
**I** **mpacts**							
**Daily functioning**				**x**			
Impaired productivity				**x**			
Avoid leisure activities^b^				**x**			
**Emotional functioning**							
Embarrassed							
Frustrated				**x**			
Depressed							
Stressed, anxiety							
**Social functioning**							
Disrupted social life (avoid social activities)				**x**			
Strained relationships							
Stigma							
**Work/school impacts**				**x**			
Absenteeism				**x**			
Presenteeism				**x**			
**Sleep disturbance**	**x**	**x**					
Difficulty falling asleep	**x**	**x**					
Difficulty staying asleep		**x**					
**Fatigue**			**x**	**x**			
Sleep disruption							
Emotional/mental			**x** ^**c**^	**x** ^**c**^			
Flares							
Treatment							
**Physical functioning**							
Avoid/limit exercise or recreational activities							
Changed hygiene routines							
Avoid moving, walking, bending^a^							

^a^Concepts mentioned only by adolescent participants

^b^Concept mentioned only by adult participants

^c^Reported by only adolescent participants

AD, atopic dermatitis; BFI-item 3, Brief Fatigue Inventory-item 3; FACIT-Fatigue, Functional Assessment of Chronic Illness Therapy-Fatigue; PGIC, Patient Global Impression of Change; PGIS, Patient Global Impression of Severity; PRO, patient-reported outcome; PROMIS-SD, Patient-Reported Outcomes Measurement Information System—Sleep Disturbance; SP-NRS, Skin Pain Numerical Rating Scale

Measures assessing fatigue (BFI-item 3 and FACIT-Fatigue) provided coverage of six concepts of interest. The BFI-item 3 covered the presence of fatigue, whereas the FACIT-Fatigue assessed how fatigue impacted daily functioning, emotional functioning, social functioning, and work/school impacts. PROMIS-SD 8a and 8b covered concepts related to sleep disturbance as described by participants in this study (Table 2).

## Discussion

This qualitative cross-sectional study examined patient experience of AD in adults and adolescents with moderate-to-severe disease. Experience of AD was found to be similar for adults and adolescents, with similar symptoms and impacts being reported. Given the evolving AD treatment landscape, the perspective of biologic-experienced patients was also considered; no differences in AD experience were observed between biologic-naïve and biologic-treated participants. Furthermore, the selected PROs were easily understood, relevant, and representative of meaningful concepts of AD for adult and adolescent participants.

According to the Global Burden of Disease study, AD carries the highest Disability-Adjusted Life-Year burden among all skin diseases, with a significantly higher burden compared with psoriasis and other skin conditions [28]. Therefore, it is important to measure patient experience of AD to evaluate whether the treatments provide meaningful benefits to patients. Before inclusion in clinical trials and practice, it is crucial to determine the PROs that measure concepts most important to patients and the appropriateness of PRO measures at assessing AD experience [8]. In line with previous research, adults and adolescents had similar experiences of AD, with itchiness and emotional functioning identified as the most bothersome AD symptoms and impacts, respectively [5, 6, 29, 30]. Notably, the current study showed some minor differences between adults and adolescents in their AD experience regarding their physical functioning, as well as attributions to fatigue. These findings highlight that although the adult and adolescent experience of AD is similar, select age-specific differences may need to be considered when selecting PROs for future AD studies.

The appropriateness of PRO measures assessing skin pain, sleep disturbance, and fatigue was evaluated, and these measures were shown to include relevant concepts that were easily understood by both adults and adolescents. Additionally, both paediatric and adult versions of measures assessing fatigue (FACIT-Fatigue) and sleep disturbance (PROMIS-SD 8a) were found to be appropriate for adolescent participants. Fatigue is not a concept routinely assessed in clinical trials of patients with AD despite being a symptom of many chronic illnesses, including asthma [31], chronic obstructive pulmonary disease [31], rheumatoid arthritis [32, 33], ​and various inflammatory dermatological conditions [14, 32, 34]. Although few participants reported fatigue spontaneously, when probed, most adults and adolescents did report that they experienced fatigue. Furthermore, most participants in this study also noted that fatigue was not something they considered a clinical aspect or symptom of their AD, but instead perceived sleep disturbance as the primary cause of their fatigue due to nocturnal scratching and picking at their skin. Although sleep disturbance is a primary driver of fatigue, fatigue also emerged as an impact for participants with AD, with fatigue also attributed to factors such as stress, anxiety, embarrassment, and AD flares. Therefore, future clinical studies should include assessments for fatigue.

Although participants identified skin pain, sleep disturbance, and fatigue as important concepts in their experience of AD, many other symptoms and impacts were reported. CE interviews captured a total of 13 symptoms and 7 impact domains experienced by adults and adolescents with AD; however, only selected instruments measuring the concepts of interest (sleep disturbance, fatigue, and pain) were tested during the CD segment of this study. While the selected PROs had not previously been validated in patients with moderate-to-severe AD, other PROs have been validated in patients with AD [18], with PROs such as the Itch Numeric Rating, Dermatology Life Quality Index and Patient-Oriented Eczema Measure capturing additional patient symptoms and impacts [22, 35, 36]. As such, additional PROs, outside of those tested here, would likely be necessary to capture the full spectrum of concepts, including all AD symptoms and their resulting impact on patients’ HRQoL.

This study was designed to align with FDA guidelines for establishing content validity for PRO measures [9]; however, there were some limitations. Firstly, the participant sample, although diverse in terms of treatment experience, was predominately White and female, and the study was conducted only on US participants, which may limit the broader applicability of the results Recent guidelines for studies assessing patients with AD have highlighted the need for a more ethnically diverse set of participants [8]. Secondly, although the study included biologic-experienced participants, sample numbers were small and saturation analyses were aggregated for biologic-naïve and biologic-experienced participants. Thirdly, despite all participants reporting moderate-to-severe AD two years prior to screening, only 2 participants self-reported either moderate-to-severe (*N* = 1) or severe (*N* = 1) AD severity at the time of screening, with many reporting their symptoms of AD as mild or under control. Therefore, reported AD severity may not reflect participants’ overall severity or that on the day of the interview. Finally, two caregivers of adolescent participants shared information or prompted participants during adolescents’ interviews, potentially influencing adolescent responses.

## Conclusions

This study enhances understanding of AD from the patient’s perspective, with minimal differences in AD experience being observed between adult or adolescent patients or between biologic-naïve and biologic-experienced patients. PRO measures for skin pain, sleep disturbance, and fatigue were found to be appropriate to use for AD in both adults and adolescents. The findings support the use of the selected PRO measures in future clinical studies.

## Electronic supplementary material

Below is the link to the electronic supplementary material.

**Supplementary Material 1**: Supplementary Figure S1. Study design. ^a^Participants grouped by biologic naïve and dupilumab experience. ^b^Half of the adolescent participants debriefed the Peds PROMIS-SD 8a and Peds FACIT-Fatigue and the other half debriefed the PROMIS-SD 8a and FACIT-Fatigue. AD, atopic dermatitis; BFI-item 3, Brief Fatigue Inventory-item 3; CD, cognitive debriefing; CE, concept elicitation; FACIT-Fatigue, Functional Assessment of Chronic Illness Therapy-Fatigue; Peds FACIT-Fatigue, Pediatric Functional Assessment of Chronic Illness Therapy–Fatigue; peds PROMIS-SD 8a, Patient-Reported Outcomes Measurement Information System—Pediatric Sleep Disturbance; PGIC, Patient Global Impression of Change; PGIS, Patient Global Impression of Severity; PRO, patient-reported outcome; PROMIS-SD, Patient-Reported Outcomes Measurement Information System—Sleep Disturbance; SP-NRS, Skin Pain Numerical Rating Scale; US, United States

**Supplementary Material 2**: Supplementary Figure S2. Body location of AD in adults and adolescents. AD, atopic dermatitis

**Supplementary Material 3**

## Data Availability

For requests for access to anonymised participant level data, please contact the Corresponding Author.

## Declarations

### Information on previous presentations

Data from this study were previously presented as poster presentations at Fall Clinical Dermatology Conference 2023, and the American Academy of Dermatology Annual Meeting (AAD), 2024.

## Abbreviations

AD: Atopic dermatitis
BFI-item 3: Brief Fatigue Inventory-item 3
CD: Cognitive debriefing
CE: Concept elicitation
FACIT-Fatigue: Functional Assessment of Chronic Illness Therapy-Fatigue
FDA: Food and Drug Administration
HRQoL: Health-related quality of life
PGIC: Patient Global Impression of Change
PGIS: Patient Global Impression of Severity
PRO: Patient reported outcome
PROMIS-SD: Patient-Reported Outcomes Measurement Information System—Sleep Disturbance
Peds FACIT-Fatigue: Pediatric Functional Assessment of Chronic Illness Therapy-Fatigue
Peds PROMIS-SD: Patient-Reported Outcomes Measurement Information System—Pediatric Sleep Disturbance
SP-NRS: Skin Pain—Numeric Rating Scale
US: United States
WCG: WIRB-Copernicus Group
