# Supplementary material for: Patient-reported outcome assessment of adults and adolescents with atopic dermatitis: a cross-sectional qualitative interview study
Source: J Patient Rep Outcomes. 2025 Apr 10;9:41. doi: 10.1186/s41687-025-00871-8 (PMC11985738; doi:10.1186/s41687-025-00871-8)
Supplement: Supplementary file 1 — Supplementary Material 1: Supplementary Figure S1. Study design. aParticipants grouped by biologic naïve and dupilumab experience. bHalf of the adolescent participants debriefed the Peds PROMIS-SD 8a and Peds FACIT-Fatigue and the other half debriefed the PROMIS-SD 8a and FACIT-Fatigue. AD, atopic dermatitis; BFI-item 3, Brief Fatigue Inventory-item 3; CD, cognitive debriefing; CE, concept elicitation; FACIT-Fatigue, Functional Assessment of Chronic Illness Therapy-Fatigue; Peds FACIT-Fatigue, Pediatric Functional Assessment of Chronic Illness Therapy–Fatigue; peds PROMIS-SD 8a, Patient-Reported Outcomes Measurement Information System—Pediatric Sleep Disturbance; PGIC, Patient Global Impression of Change; PGIS, Patient Global Impression of Severity; PRO, patient-reported outcome; PROMIS-SD, Patient-Reported Outcomes Measurement Information System—Sleep Disturbance; SP-NRS, Skin Pain Numerical Rating Scale; US, United States [file 41687_2025_871_MOESM1_ESM.pdf]

Screening

- US-based
- Able to speak and read English
- Self-reported moderate-to-severe symptoms of AD within the past 2 years
- Confirmed AD diagnosis
- Proof of oral corticosteroid or biologic use within the past 2 years (via medical record or prescription) as a proxy for severity[a]
- Willing and able to participate in a 90-minute interview

→

90-minute interview

Concept Elicitation (CE):
Open-ended questions to elicit descriptions of symptoms, impacts and overall experience of AD

Cognitive Debriefing (CD):
Completion of each PRO measure using a think-aloud method and questions on comprehension, relevance and interpretation of each **PRO measure**

- PROMIS-SD 8a/b & PROMIS Pediatric 8a & 8b
- BFI-Item 3
- FACIT-Fatigue & Peds FACIT-Fatigue
- SP-NRS
- PGI-S
- PGI-C

→

Data analyses
Audio-recorded interviews were transcribed, quality checked, and CE and CD data were coded and analyzed

**PROMIS-SD 8a/8b, PROMIS Pediatric 8a[b]**	Assessment of sleep disturbance due to AD over the past 7 days *[5-point Likert scales]*
**BFI-Item 3**	Worst level of fatigue during the past 24 hours *[0–10 numeric scale]*
**FACIT- Fatigue, Peds FACIT-Fatigue[b]**	Assessment of fatigue over the past 7 days *[5-point Likert scales]*
**SP-NRS**	Worst level of skin pain in the past 24 hours *[0–10 numeric scale]*
**PGI-S**	Patient global impression of severity of skin pain over the past 7 days *[5-point Likert scale]*
**PGI-C**	Patient global impression of change in skin pain now, as compared with 7 days ago *[7-point Likert scale]*
