# Supplementary material for: Patient-reported outcome assessment of adults and adolescents with atopic dermatitis: a cross-sectional qualitative interview study
Source: J Patient Rep Outcomes. 2025 Apr 10;9:41. doi: 10.1186/s41687-025-00871-8 (PMC11985738; doi:10.1186/s41687-025-00871-8)
Supplement: Supplementary file 3 — Supplementary Material 3 [file 41687_2025_871_MOESM3_ESM.docx]

*Supplementary Table S1. Interview guide overview*

| Interview section | Topics covered |
| --- | --- |
| 1. Introduction and background | - Greeting and introductions between the researcher and participant - Brief background on the project and explanation of the purpose of the interview - Review of consent and permission to audio-record interview |
| 1. CE | - Open-ended questions regarding the participant’s experience of:   - AD symptoms   - Impacts related to AD |
| 1. CD | - Questionnaires completed by the participant using the think-aloud approach - Assessment of the relevance of concepts, comprehensiveness, and comprehensibility of items, and appropriateness of the response options and recall period - Additional discussion on any parts of the measure(s) the participant found confusing - Discussion on meaningful change – symptom resolution and perceived success treatment outcome |
| 1. Conclusion | - Any additional information the participant had not already shared - Discussion about honorarium |

AD, atopic dermatitis; CD, cognitive debriefing; CE, concept elicitation.

*Supplementary Table S2. Self-reported AD severity on the day of screening^a^*

| Current severity  n (%) | Adults | | | Adolescents | | |
| --- | --- | --- | --- | --- | --- | --- |
|  | **Biologic-naïve**  **(N=8)** | **Dupilumab- experienced (N=8)** | **Total  (N=16)** | **Biologic-naïve**  **(N=10)** | **Dupilumab- experienced (N=10)** | **Total**  **(N=20)** |
| Severe | 0 | 0 | 0 | 0 | 1 (10) | 1 (5) |
| Moderate-to-severe | 0 | 0 | 0 | 0 | 1 (10) | 1 (5) |
| Moderate | 4 (50) | 2 (25) | 6 (38) | 4 (40) | 2 (20) | 6 (30) |
| Mild | 2 (25) | 4 (50) | 6 (38) | 3 (30) | 4 (40) | 7 (35) |
| Under control/better | 2 (25) | 6 (75) | 8 (50) | 2 (20) | 4 (40) | 6 (30) |

^a^ Inclusion criteria stipulated that participants must have physician-diagnosed AD and self-reported experiencing moderate-to-severe AD symptoms within the last two years.
AD, atopic dermatitis.

|  | **Adults** | | | | **Adolescents** | | | | |
| --- | --- | --- | --- | --- | --- | --- | --- | --- | --- |
| **Group number (number of interviews)** | Group 1  (1–4) | Group 2  (5–8) | Group 3  (9–12) | Group 4  (13–16) | Group 1  (1–4) | Group 2  (5–8) | Group 3  (9–12) | Group 4  (13–16) | Group 5  (17–20) |
| **Total AD severity identified in each set** | 4 | 1 | 0 | 0 | 2 | 2 | 2 | 0 | 0 |
| **Total body location identified in each set** | 4 | 0 | 0 | 0 | 4 | 1 | 0 | 0 | 0 |
| **Total symptoms identified in each set** | 12 | 0 | 0 | 0 | 11 | 1 | 0 | 0 | 0 |
| **Total triggers identified in each set** | 12 | 3 | 0 | 0 | 7 | 4 | 1 | 2 | 0 |
| **Total impact domains identified in each set** | 7 | 0 | 0 | 0 | 7 | 0 | 0 | 0 | 0 |
| **Total concepts identified in each set** | 38 | 4 | 0 | 0 | 31 | 8 | 3 | 2 | 0 |
| **Percentage of concepts identified in each set** | 90% | 10% | 0% | 0% | 70% | 18% | 7% | 5% | 0% |
|  | | | | | | | | | |
| **Total codes identified** | 42 | 0 | 0 | 0 | 31 | 39 | 42 | 44 | 44 |

*Supplementary Table S3. Saturation grid^a^*

^a^Concepts included in saturation analyses were those probed in the interview guide, as well as any themes which emerged naturally during the interview; concepts of interest included severity of AD, body location of AD, symptoms, triggers, and impact domains
AD, atopic dermatitis

| Triggers, Exacerbations n (%) | Adults | | | | Adolescents | | | |
| --- | --- | --- | --- | --- | --- | --- | --- | --- |
|  | **Biologic-naïve  (N=8)** | **Dupilumab- experienced (N=8)** | **Total (N=16)** | **Biologic-naïve (N=10)** | | **Dupilumab- experienced (N=10)** | **Total (N=20)** |  |
| Stress | 1 (13) | 5 (63) | 6 (38) | 2 (20) | | 0 | 2 (10) |  |
| Consumer Products |  |  |  |  | |  |  |  |
| Fragrance | 1 (13) | 3 (38) | 4 (25) | 2 (20) | | 1 (10) | 3 (15) |  |
| Soaps and lotions | 2 (25) | 5 (63) | 7 (44) | 3 (30) | | 4 (40) | 7 (35) |  |
| Cleaning products | 2 (25) | 3 (38) | 5 (31) | 0 | | 1 (10) | 1 (5) |  |
| Clothing | 1 (13) | 3 (38) | 4 (25 | 0 | | 3 (30) | 3 (15) |  |
| Food/diet | 1 (13) | 2 (25) | 3 (19) | 0 | | 1 (10) | 1 (5) |  |
| Environment/Weather |  |  |  |  | |  |  |  |
| Allergies | 1 (13) | 1 (13) | 2 (13) | 0 | | 2 (20) | 2 (10) |  |
| Sun | 3 (38) | 1 (13) | 4 (25) | 2 (20) | | 0 | 2 (10) |  |
| Heat | 2 (25) | 0 | 4 (25) | 4 (40) | | 4 (40) | 8 (40) |  |
| Water | 1 (13) | 3 (38) | 4 (25) | 4 (40) | | 3 (30) | 7 (35) |  |
| Being complacent | 0 | 2 (25) | 2 (13) | 0 | | 1 (10) | 1 (5) |  |
| Sweat | 1 (13) | 1 (13) | 2 (13) | 3 (30) | | 4 (40) | 7 (35) |  |
| Itching/scratching | 0 | 2 (25) | 2 (13) | 1 (10) | | 0 | 1 (5) |  |

*Supplementary Table S4. Triggers and exacerbations for adults and adolescents with AD*

AD, atopic dermatitis.

*Supplementary Table S5. Breakdown of AD impacts for adults and adolescents*

| Impacts  n (%) | Adults | | | Adolescents | | |
| --- | --- | --- | --- | --- | --- | --- |
|  | Biologic-naïve  (N=8) | Dupilumab- experienced (N=8) | Total  (N=16) | Biologic-naïve  (N=10) | Dupilumab- experienced (N=10) | Total  (N=20) |
| Emotional functioning | **7 (88)** | **8 (100)** | **15 (94)** | **10 (100)** | **9 (90)** | **19 (95)** |
| Embarrassed | 6 (75) | 7 (88) | 13 (81) | 10 (100) | 8 (80) | 18 (90) |
| Frustrated | 1 (13) | 4 (50) | 5 (31) | 1 (10) | 4 (40) | 5 (25) |
| Depressed | 2 (25) | 3 (38) | 5 (31) | 1 (10) | 3 (30) | 4 (20) |
| Stressed, anxiety | 2 (25) | 3 (38) | 5 (31) | 3 (30) | 3 (30) | 6 (30) |
| Fatigue | **8 (100)** | **7 (88)** | **15 (94)** | **6 (60)** | **7 (70)** | **13 (65)** |
| Stress, anxiety, embarrassment | 3 (38) | 4 (50) | 7 (44) | 3 (30) | 1 (10) | 4 (20) |
| Sleep disruption | 4 (50) | 3 (38) | 7 (44) | 4 (40) | 6 (60) | 10 (50) |
| Flares | 0 | 0 | 0 | 1 (10) | 2 (20) | 3 (15) |
| Sleep disturbance | **7 (88)** | **7 (88)** | **14 (88)** | **6 (60)** | **9 (90)** | **15 (75)** |
| Difficulty staying asleep | 5 (63) | 6 (75) | 11 (69) | 4 (40) | 6 (60) | 10 (50) |
| Difficulty falling asleep | 3 (38) | 4 (50) | 7 (44) | 6 (60) | 9 (90) | 15 (75) |
| Social functioning | **6 (75)** | **8 (100)** | **14 (88)** | **9 (90)** | **8 (80)** | **17 (85)** |
| Disrupted social life | 3 (38) | 4 (50) | 7 (44) | 5 (50) | 4 (40) | 9 (45) |
| Stigma | 4 (50) | 3 (38) | 7 (44) | 7 (70) | 5 (50) | 12 (60) |
| Strained relationships | 3 (38) | 1 (13) | 4 (25) | 2 (20) | 2 (20) | 4 (20) |
| Daily functioning | **3 (38)** | **7 (88)** | **10 (63)** | **7 (70)** | **6 (60)** | **13 (65)** |
| Impaired productivity | 3 (38) | 3 (38) | 6 (38) | 7 (70) | 6 (60) | 13 (65) |
| Avoid or limit leisure activities | 0 | 2 (25) | 2 (13) | 0 | 0 | 0 |
| Work/school impacts | **3 (38)** | **5 (63)** | **8 (50)** | **6 (60)** | **8 (80)** | **14 (70)** |
| Presenteeism | 2 (25) | 3 (38) | 5 (31) | 5 (50) | 5 (50) | 10 (50) |
| Absenteeism | 2 (25) | 1 (13) | 3 (19) | 4 (40) | 7 (70) | 11 (55) |
| Physical functioning | **4 (50)** | **2 (25)** | **6 (38)** | **9 (90)** | **9 (90)** | **18 (90)** |
| Avoid/limit exercise or recreational activities | 3 (38) | 1 (13) | 4 (25) | 5 (50) | 5 (50) | 10 (50) |
| Changed hygiene routine | 2 (25) | 1 (13) | 3 (19) | 7 (70) | 9 (90) | 16 (80) |
| Avoid movement | 0 | 0 | 0 | 1 (10) | 2 (20) | 3 (15) |

AD, atopic dermatitis.

*Supplementary Table S6. Exemplary quotes from CD on select PRO measures from adults and adolescents*

| PRO measure | Adult exemplary quote | Adolescent exemplary quote |
| --- | --- | --- |
| PROMIS-SD 8a & b  (and Peds PROMIS SD 8a for adolescents) | Sometimes… I go to sleep and then three or four hours later, you know, I wake up and I notice that I’m—I’m itching, you know, and I’m hurting myself. And I wake up and I put—I put cream on it, and then I’ll try to go to sleep again. (Adult 015) | Well, it is relatable, um, because it describes, you know, a lifestyle standpoint and how this problem is affecting me. So I think it's, you know, excellent. (Adolescent 046 on the PROMIS-SD 8a)  I feel like it was easy to decide, like, based on recent, like, symptoms and experiences and it wasn't confusing or anything. (Adolescent 039 on the Peds PROMIS SD 8a) |
| BFI-item 3 | Sometimes I get really tired you know, and I can’t get to do things I want to do, so I kind of put them off. (Adult 021) | Um, I think it’s relevant all the time…Uh, it’s probably more, um—what’s the word—Like, more applies to me, um, when I have a flare-up, because that’s typically when I feel more fatigued…Because my body is just, like, tired of it, I guess…But, um, I think it does affect me all the time. (Adolescent 042) |
| FACIT-Fatigue  (and Peds FACIT-Fatigue for adolescents) | I have felt tired before because when I was having, er—flare-ups, severe ones, it—it kept me up and my sleep pattern was screwed, and I was fatigued in the morning. And I was a little—yeah, emotional, you know. (Adult 022) | Yeah, I actually thought this one was like, um, more like applied to my experience. The questions kind of like related more like needing help with things. Like I know sometimes when it was like really bad. Like when I was younger like my mom would like help me like shower, like do my hair, like stuff like that. So, if it was like really bad again, like extremely severe, those kind of questions are more, uh, like helpful. (ADOL050 on the FACIT-Fatigue)  I definitely think it's like a good insight to see how like people who struggle with fatigue due to AD like go about their daily lives and how much it affects them within like a week span time. (Adolescent 044 on the peds FACIT-Fatigue) |
| SP-NRS | I like it. I think 10, 0 to 10 is a great score. Um—and I like—I like how it says no pain or worst pain imaginable. So, um—I like—I like that, um—um—there are good comparisons there. They are level, you know. (Adult 021) | Um, yeah, I think so. Um, especially like during a flare-up when I do have like more pain and discomfort because of my AD. (Adolescent 044) |
| PGIS | I would say moderate… I had to think about it because, er—like my fingers are not bothering me at the moment but the inside of my hands is… I was thinking, um—is it for one area or for the whole thing, the severity? So, I had to think about it. I was zeroing on the parts that was um—feeling the most pain. (Adult 028) | Uh, yeah, because I definitely have like some discomfort and stuff and especially when I have a flare-up. (Adolescent 044) |
| PGIC | I would say it's, it’s makes sense. It gives you plenty of options. Um—either you, it was very much improved or very much worse, or you could pick in between.  (Adult 023) | Uh yeah, I would say so because like I definitely like can tell when my discomfort gets worse. (Adolescent 044) |

AD, atopic dermatitis; BFI-item 3, Brief Fatigue Inventory-item 3; CD, cognitive debriefing; FACIT-Fatigue, Functional Assessment of Chronic Illness Therapy-Fatigue; peds FACIT-Fatigue, Pediatric FACIT-Fatigue; peds PROMIS-SD 8a, Patient-Reported Outcomes Measurement Information System—Pediatric Sleep Disturbance; PGIC, Patient Global Impression of Change; PGIS, Patient Global Impression of Severity; PRO, patient-reported outcome; PROMIS-SD, Patient-Reported Outcomes Measurement Information System—Sleep Disturbance; SP-NRS, Skin Pain Numerical Rating Scale.
